# Hybrid *De Novo* Genome Assembly Using MiSeq and SOLiD Short Read Data

**DOI:** 10.1371/journal.pone.0126289

**Published:** 2015-04-28

**Authors:** Tsutomu Ikegami, Toyohiro Inatsugi, Isao Kojima, Myco Umemura, Hiroko Hagiwara, Masayuki Machida, Kiyoshi Asai

**Affiliations:** 1 Information Technology Research Institute, National Institute of Advanced Industrial Science and Technology (AIST), Tsukuba, Ibaraki, Japan; 2 Cift Corporation, Tsukuba, Ibaraki, Japan; 3 Bioproduction Research Institute, National Institute of Advanced Industrial Science and Technology (AIST), Sapporo, Hokkaido, Japan; 4 Computational Biology Research Center, National Institute of Advanced Industrial Science and Technology (AIST), Tokyo, Japan; CNRS UMR7622 & University Paris 6 Pierre-et-Marie-Curie, FRANCE

## Abstract

A hybrid *de novo* assembly pipeline was constructed to utilize both MiSeq and SOLiD short read data in combination in the assembly. The short read data were converted to a standard format of the pipeline, and were supplied to the pipeline components such as ABySS and SOAPdenovo. The assembly pipeline proceeded through several stages, and either MiSeq paired-end data, SOLiD mate-paired data, or both of them could be specified as input data at each stage separately. The pipeline was examined on the filamentous fungus *Aspergillus oryzae* RIB40, by aligning the assembly results against the reference sequences. Using both the MiSeq and the SOLiD data in the hybrid assembly, the alignment length was improved by a factor of 3 to 8, compared with the assemblies using either one of the data types. The number of the reproduced gene cluster regions encoding secondary metabolite biosyntheses (SMB) was also improved by the hybrid assemblies. These results imply that the MiSeq data with long read length are essential to construct accurate nucleotide sequences, while the SOLiD mate-paired reads with long insertion length enhance long-range arrangements of the sequences. The pipeline was also tested on the actinomycete *Streptomyces avermitilis* MA-4680, whose gene is known to have high-GC content. Although the quality of the SOLiD reads was too low to perform any meaningful assemblies by themselves, the alignment length to the reference was improved by a factor of 2, compared with the assembly using only the MiSeq data.

## Introduction

Thanks to the rapid development of the next-generation sequencing (NGS) technologies, the DNA sequencing throughput is increasing much faster than CPU performance. The NGS platforms, such as the Illumina HiSeq/MiSeq systems and the Applied Biosystems SOLiD system, produce millions to billions of short read data routinely. Consequently, efficient tools to handle such massive amounts of data become necessary, especially for the de novo whole genome assembly. To reconstruct genome sequences from short read data, an algorithm based on the de Bruijn graph representation [1] was shown to be efficient. A number of assembly tools using this algorithm were thus developed and are publicly available now [2]. Unfortunately, the format and semantics of the NGS data varies depending on the biochemical protocols employed and few tools are capable of handling different types of short read data in a hybrid manner [3–6].

In a previous report [7], we have shown that a fungal genome can be assembled by solely using SOLiD short read data of 50 bp length each, with an N50 value of 900 kbp. Since then, several NGS assembly studies of fungal species were reported [8–12], where N50 values range from 50 kbp to 500 kbp. Among them, the detailed comparison of the assembly results with the reference genome sequence [7] reveals that 99% of genes on the reference were successfully located, though the average length of base pairs aligned consecutively on the reference was at most 30 kbp. One of the major topics in fungal genomics is to discover genes relating to secondary metabolite biosyntheses (SMB). Because a cluster of SMB genes typically extends over more than 50 kbp range, an assembly method that gives a longer alignment to the reference is required.

Recently, we found that the alignment length can be improved by using both MiSeq and SOLiD short read data appropriately during the de novo assembly process. In the next section, an outline of our assembly pipeline is introduced. The assessment of the pipeline is described in the subsequent section using filamentous fungus *Aspergillus oryzae* RIB40 and actinomycete *Streptomyces avermitilis* MA-4680 as benchmarks.

## Methods and Materials

### Hybrid assembly pipeline

**Fig 1:**
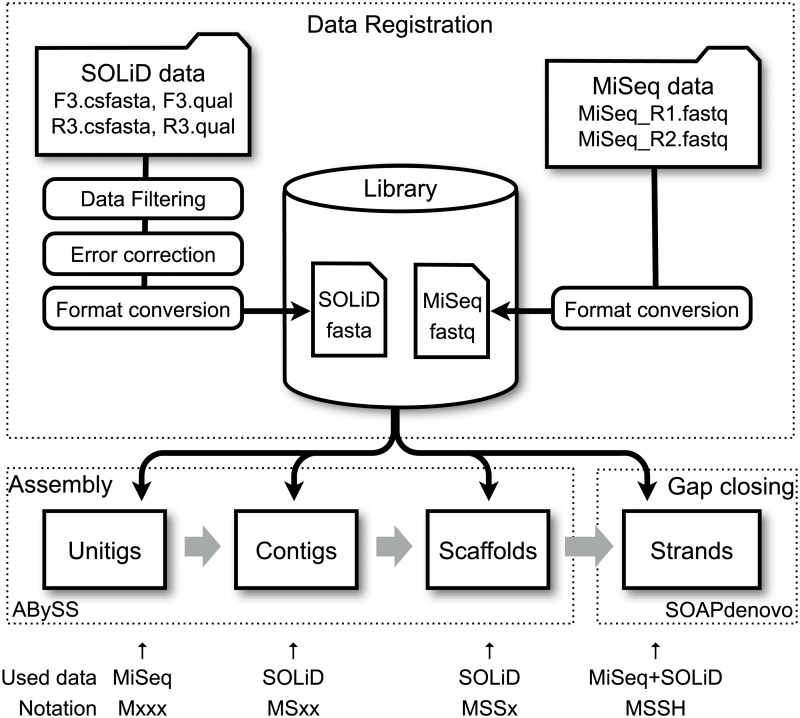
Overview of the hybrid assembly pipeline. Raw data generated by several NGS platforms are preprocessed into a common format, which is registered to the library. A data set used at each assembly stage can be specified separately. The assembly results are denoted according to the supplied data, as illustrated at the bottom of the figure.

A hybrid assembly pipeline was constructed to utilize both MiSeq and SOLiD short read data in combination. The pipeline is composed of three procedures: data registration, assembly, and gap closing. A diagram of the pipeline is shown schematically in Fig 1.

In the data registration procedure, raw short read data from MiSeq and SOLiD platforms are sanitized and recast in the common, standard FASTA/FASTQ format with the following constraints for the mate-paired and paired-end data:

- Paired data are merged into a single file in an interleaved manner.
- Tags for the paired entries are postfixed by “/1” and “/2”.
- Paired entries are converted to the forward-reverse (FR) orientation. That is, both 5’-ends of a DNA fragment and its complement are paired.

For the MiSeq paired-end data, the raw short read data are provided in two FASTQ files for forward and reverse reads, which are simply stitched together after tag modification. The processing of the SOLiD mate-paired data is more involved. SOLiD mate-paired data are provided in a XSQ file [13], which is decomposed into two pairs of files by XSQ tools (ver. 1.5) [14]. Each pair consists of a short read file and a quality file. The short read data are then down-sampled referring to the quality values [7] as follows. For each short read, the number of color calls with quality values lower than 10 (i.e., less than 90% accuracy) is counted. If the number exceeds a threshold value *N*_lowQ_, the corresponding short read is omitted along with its mated counterpart. The *N*_lowQ_ value was taken such that the coverage of the SOLiD reads become about 120. No further filtering, such as adapter filtering, was performed, because the prepared DNA samples were long enough to avoid sequencing of the adapter region. The down-sampled data are subjected to error correction by the SOLiD Accuracy Enhancement Tool (SAET) by Applied Biosystems [15, 16]. At this point, the pair of short read data are encoded in the 2 base color codes (color space) [17], and are in the forward-forward (FF) orientation, where two 5’ to 3’ reads of the same DNA fragment are paired [18]. They are thus converted into the base space and transformed into FR orientation, followed by tag modification and stitching. Note that, because of the conversion from the color space to the base space, a single read error makes the subsequent portion of the short read in the base space completely unusable. Therefore, the SOLiD data are less reliable under the read error situation, as far as the present approach requiring the conversion is employed.

In the assembly procedure, ABySS (ver. 1.3.4) [19] is used to compile short read data into scaffolds. Assembly in ABySS proceeds in three stages: formation of unitigs, contigs, and scaffolds. At the unitig stage, short read data is decomposed into k-mers, the de Bruijn graph is constructed and cleaned up; and the Euler path problem is solved to give assembled sequences as unitigs. These unitigs are then linked with each other by using the paired-end/mate-paired information to create contigs. In this contig stage, paired short reads are mapped onto the unitigs, in order to obtain adjacent information between unitigs, as well as an estimate of the insertion length between pairs. For this mapping procedure, we used the KAligner program of ABySS with the same k-mer size parameter used to construct the de Bruijn graph. At the scaffolding stage, the contigs are further linked together by using the paired information. The resulting scaffolds have multiple gaps, where a sequence of “N”s are inserted at undetermined parts of the sequences.

In the gap closing procedure, these gaps are filled by the GapCloser program of SOAPdenovo [20]. Hereafter, we refer to the resulting gap filled sequences as strands.

At each stage to construct unitigs, contigs, scaffolds, and strands, we can select a set of short read data to be used. Here, we employ the notation “M”, “S”, and “H” for the MiSeq, SOLiD, and Hybrid (MiSeq + SOLiD) data, respectively, and concatenate them to denote the combination of data sets used in the assembly. For example, if only MiSeq data were utilized at all stages of the assembly, this would be denoted as MMMM. If we construct unitigs with MiSeq data (M), contigs and scaffolds using SOLiD data (S), and strands with both MiSeq and SOLiD data (H), this would be denoted as MSSH. In this paper, various assembly modes of the pipeline were compared and analyzed.

The hybrid assemblies were processed on a cluster computer. Each computation node is equipped with two Intel Xeon E5-2620 CPUs (2 GHz, 6 cores) and 64 GB of memory, and interconnected by 10GbEthernet. Typically, 4 ∼ 8 nodes are utilized for the unitig stage, while the remaining stages are processed on a single node. The scalable implementation of ABySS allows the pipeline to assemble about 130 Gbp reads at one time if sufficient number of computation nodes are available.

### Assessment of assembled sequences

The assembled sequences are compared with the reference sequences to assess the performance of the assembly. Before the assessment, the obtained strands are processed as follows. The strands are first filtered by length where those sequences shorter than arbitrarily 500 bp are removed. The filtered strands are then mapped to a set of known sequences of the GenBank nucleotide collection (nt) by the Blastn program [21, 22] to eliminate trivial contaminants. In the present analysis, the *A. oryzae* sample was found to be contaminated by *B. subtilis*, so that those strands matching *B. subtilis* with e-values lower than 10^−100^ were removed. No noticeable contamination was found for *S. avermitilis*.

The resulting set of strands is used in the performance assessment, which is based on the following three criteria. First, the global reproducibility of the nucleotide sequence is assessed by the R50 value [7]. To calculate R50, strands are mapped onto the reference by the LAST program [23, 24], and the reference sequences are broken into fragments such that each fragment is aligned to one of the strands. The total length of the fragments should be almost the same as those of the reference, if the whole reference is covered by the strands. The R50 value is calculated as N50 of the fragments, i.e., the sum of the length of the fragments longer than R50 becomes the half of the total length. Therefore, the more successful the assembly, the larger the R50 value will be. Note that, different from the N50 value, R50 is not affected by contaminants, because only those strands aligned to the reference are used in the calculation of R50.

Second, the reproducibility of gene-coding regions is assessed by locating the open reading frames (ORFs) on the strands. The ORF sequences on the reference are mapped on the strands by the LAST program, and the alignment with the best score is selected for each ORF. The number of aligned ORFs is counted according to their alignment qualities such as the number of ORFs aligned identically, the number aligned with high-score (e-values < 10^−100^), and others.

Third, the integrity of gene clusters is assessed by mapping SMB gene clusters on the strands. For each SMB cluster, a minimal nucleotide sequence that covers all the ORFs in the cluster are extracted from the reference, and is mapped on the strands by LAST. The number of identically aligned SMB clusters is examined, as well as the number of well-aligned clusters, where 99.9% of the nucleotide sequence is recovered from at most two strands. This is the most stringent test of the performance of the assembly because intergenic sequences are included and because the majority of SMB clusters are located at the subtelomeric region with many repeated sequences expected.

### Strain, medium and DNA preparation

The fungal strain, *A. oryzae* RIB40, obtained from the National Research Institute of Brewing, Japan (http://www.nrib.go.jp/ken/asp/strain.html), was grown in liquid YPD (Yeast extract, Peptone, Dextrose) medium (Difco) at 30°C for 2 days. Genomic DNA was isolated from RIB40 according to the method described by Umemura *et al*. [7].

The bacterial strain, *S. avermitilis* MA-4680, obtained from National Institute of Technology and Evaluation, Japan (http://www.nite.go.jp), was grown in ATCC Medium 1877: ISP Medium 1 (5 g of tryptone and 3 g of Yeast extract in 1 L medium, pH 7.0–7.2) at 28°C for 2 days. Cells were harvested by centrifugation and ground into fine powder in the presence of liquid nitrogen. Genomic DNA was recovered using Masterpure Yeast DNA purification kit (epicentre) according to the manufacturer’s instruction followed by 2-propanol precipitation. The DNA was then dissolved in TE at 4°C and subjected to RNase treatment (10 mg/ml for 1 h at 37°C followed by proteinase K treatment (10 mg/ml for 2 h at 55°C. After phenol/chloroform extraction, the DNA was precipitated with 2-propanol and dissolved in TE.

### Whole genome sequencing

The SOLiD reads for *A. oryzae* were obtained from the mate-paired library lib1.9 [7], which were derived from sheared genomic DNA fragments of 1.9 kbp in average size. The SOLiD reads for *S. avermitilis* were obtained from a mate-paired library harboring sheared genomic DNA fragments of 1.5 kbp in average size. The MiSeq reads were obtained from paired-end libraries containing DNA fragments of 0.5 kbp in average size, prepared by using Nextera DNA Sample Preparation Kit, for both *A. oryzae* and *S. avermitilis*.

## Results and discussions

### *Aspergillus oryzae* RIB40

**Properties of the short read data** The base information of the input short read data for *A. oryzae* assemblies is summarized in Table 1. The SOLiD data was down-sampled at *N*_lowQ_ = 25, statistics of which is also listed in Table 1. Note that the SOLiD data were generated without the Exact Call Chemistry (ECC) module [25], so that they may be less accurate. The reference sequences [26] and a set of 11902 ORFs were obtained from [27]. The location of the SMB gene clusters was taken from [28], where 75 candidates were listed.

**Table 1:** Statistical information of input short read data.

	insertion length^a^	read length	number of pairs	total bp
*A. oryzae*				(37 Mbp)^b^
MiSeq	292 ± 140	221 ± 56	3.9M	1.7 Gbp
SOLiD	–	50	51M	5.1 Gbp
qv25^c^	1666 ± 268	50	49M	4.9 Gbp
*S. avermitilis*				(9.0 Mbp)^b^
MiSeq	284 ± 137	222 ± 55	3.9M	1.7 Gbp
SOLiD	–	50	81M	8.1 Gbp
qv6^c^	1093 ± 200	50	9.8M	0.98 Gbp

^a^ Insertion length is estimated by mapping paired reads on the assembled results.

^b^ Total length of the reference sequences [26, 29].

^c^ SOLiD data are down-sampled at *N*_lowQ_ = 25 (qv25) and 6 (qv6).

**Global reproducibility** Characteristics of the strands for *A. oryzae*, including the N50 value, the maximum length, and the R50 value, are summarized in Table 2 for the hybrid assemblies (HHHH, MSSH), the MiSeq only assembly (MMMM), and the SOLiD only assembly (SSSS). The assembly was performed with various k-mer sizes, and the most optimal k-mer size was selected based on the R50 values of unitigs (Table 3). Note that, because the read length of the SOLiD data is 50 bp, the k-mer size must be less than 50 for the SOLiD and hybrid assemblies. The results from our previous assembly pipeline [7], denoted as denovo2, is also listed in Table 2. Although both the SSSS and the denovo2 assemblies use the same SOLiD data, denovo2 gives a better result because it is processed in the color space, and is more robust against read errors.

**Table 2:** Characteristic indices of the strands from several assemblies.

mode	k-mer size	Number	N50 (kbp)	Max (kbp)	R50 (kbp)
*A. oryzae*					
HHHH	45	145	1301	3191	221
MSSH	45	145	1344	3291	210
MMMM	51	1109	74.2	316	65.3
SSSS	33	1141	235	1008	15.9
denovo2	31	153	924	1957	26.5
*S. avermitilis*					
HHHH	45	90	486	1319	198
MSSH	45	96	493	1436	148
MMMM	51	233	110	511	99.2
SSSS	33	2821	6.44	56.4	0.535
denovo2	31	808	31.0	154	0.882

**Table 3:** K-mer size dependence of the R50 values^a^ of the *A. oryzae* ^b^ unitigs.

k-mer size	21	27	33	39	45	51	57
Hxxx	3.15	7.99	22.9	44.0	52.9	–	–
Mxxx	6.01	23.5	32.0	36.8	40.8	43.6	43.6
Sxxx	2.09	3.48	4.15	3.61	1.26	–	–

^a^ in kbp unit.

^b^ *S. avermitilis* results are omitted due to the erroneous SOLiD reads.

**Fig 2:**
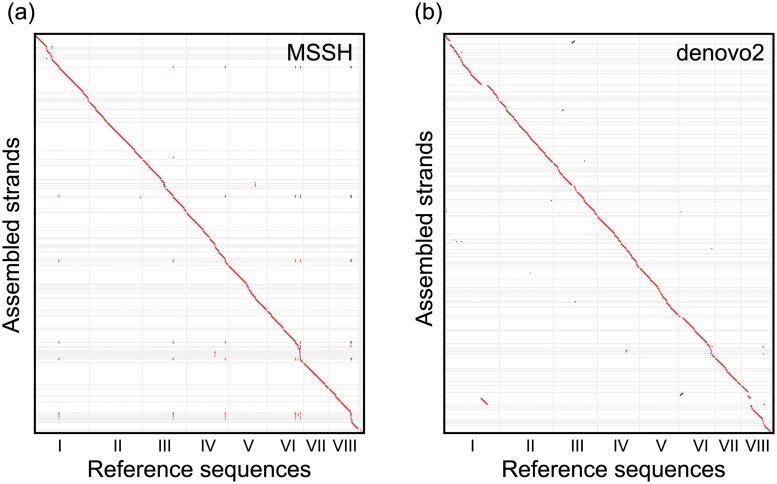
Dotplot alignments of assembled strands against the reference genome sequence of *A. oryzae*. Alignments shorter than 4000 bp were omitted from the plots. Forward and reverse alignments are plotted in red and blue colors, respectively. The Roman numerals I-VIII on the abscissa are the chromosome index of *A. oryzae*. (a) The MSSH assembly, (b) the denovo2 assembly.

As shown in Table 2, the quality of the assemblies is improved by using both the MiSeq and the SOLiD data in a hybrid manner. The N50 and R50 values of MSSH are 18 and 3 times larger than that of MMMM, respectively, indicating that the hybrid assembly produces much longer strands than the MiSeq only assembly. Similarly, the MSSH demonstrates better reproducibility of the nucleotide sequences than the SOLiD only assembly with N50 and R50 values that are 8 and 1.5 times larger than that of denovo2. To compare the MSSH and denovo2 assemblies graphically, dotplots were drawn against the reference sequences (Fig 2). Alignments shorter than 4000 bp were omitted in the plots. A few major misjoins are noticeable in denovo2, especially at chromosome VIII that disappear in MSSH. Several minor misjoins can be seen in MSSH, which are located at telomeric areas (IV, VI, VIII) and regions with the high AT content (I, III). Based on the discussions in the latter sections, we suspect that these misjoins are caused by a lack of reliable SOLiD data around these regions.

In the MSSH assembly, we employ the MiSeq data at the unitig stage, the SOLiD data at the contig/scaffolding stages, and both data at the gap closing stage. The MSSH hybrid mode was selected for *A. oryzae* by the following procedures. First, as shown in Table 3, the R50 values of the unitigs constructed from the MiSeq data (denoted as Mxxx) are better than the SOLiD only results (Sxxx), in part due to the longer read length of MiSeq. The R50 values of the unitigs constructed from both the MiSeq and the SOLiD data (Hxxx) is between Mxxx and Sxxx for smaller k-mer sizes, and then improves over Mxxx for larger k-mer sizes. Note that the number of k-mers enrolled in the de Bruijn graph is decreased with the k-mer size, whose rate is proportional to the number of short reads. In the present case, the number of k-mers from the MiSeq data exceeds that from the SOLiD data at the k-mer size of 33. That is, the construction of the Hxxx unitigs at the larger k-mer sizes are mainly driven by the MiSeq data, with some help from the SOLiD data. As will be discussed in the next section, incorporation of the SOLiD data may induce misassemblings at gene regions with high AT contents [30], despite the overall improvement of the reproducibility. To be conservative, our preference is to use only the MiSeq data at the unitig stage, at least for the SOLiD data without the ECC module.

Characteristics of the contigs, scaffolds, and strands for *A. oryzae* are summarized in Table 4. Starting from the MiSeq unitigs, either the MiSeq (MMMx), the SOLiD (MSSx), or both of the data (MHHx) are applied in the contig/scaffold stages. Apparently, the inclusion of the SOLiD data is essential at these stages: the N50 values and the maximum lengths indicate that the MSSx scaffolds are longer than the MMMx ones by a factor of more than 10. The longer insertion length of the SOLiD mate-paired reads (∼ 1600 bp) seems to be helpful in bridging unitigs. Note that the R50 value was improved by 2 kbp by including the MiSeq data additionally at the contig stage. This improvement, however, can also be recovered at the gap closing stage, so that we need only to rely on the SOLiD data to construct contigs/scaffolds. The choice of the data set at the gap closing stage was also assessed and is summarized in Table 4. Starting from the MSSx scaffolds, either the MiSeq (MSSM), the SOLiD (MSSS), or both of the data (MSSH) are used for the gap closing. Judging from the R50 values, both the MiSeq and the SOLiD data benefit the quality of the final strands, so that we employ both of the data in the gap closing stage.

**Table 4:** Characteristics of the *A. oryzae* ^a^ contigs/scaffolds/strands from several assemblies^b^.

mode	N50 (kbp)	Max (kbp)	R50 (kbp)
Contigs			
MHxx	281	821	88.5
MMxx	70.0	316	62.7
MSxx	281	821	86.5
Scaffolds			
MHHx	1345	3291	88.5
MMMx	74.2	316	62.7
MSSx	1345	3291	86.5
Strands			
MSSH	1344	3291	210
MSSM	1344	3291	159
MSSS	1345	3291	183

^a^ *S. avermitilis* results are omitted due to the erroneous SOLiD reads.

^b^ k-mer size is fixed at 45.

**Reproducibility of gene-coding regions** The number of ORFs reproduced in the assembled strands is summarized in Table 5. The MSSH assembly reproduced 98.6% of ORFs identically, and the remainder of the ORFs are aligned with high-score (e-values < 10^−100^). The good reproducibility may originate from the MiSeq data: all the ORFs are already found in the Mxxx unitigs (k-mer size = 45), with 96.8% of identical alignments.

**Table 5:** Number of ORFs reproduced in the assemblies.

mode	identical	high-score^a^	matched	missing
*A. oryzae*	(11902)^b^			
HHHH	11749	144	4	5
MSSH	11737	165	0	0
MMMM	11591	307	4	0
SSSS	8527	3328	32	15
denovo2	9908	1972	15	7
*S. avermitilis*	(7683)^b^			
HHHH	7346	318	19	0
MSSH	7344	321	18	0
MMMM	7331	331	21	0
SSSS	550	2924	2877	1332
denovo2	1578	4478	1523	104

^a^ Number of ORFs aligned with e-values lower than 10^−100^.

^b^ Total number of ORFs.

On the other hand, in the HHHH assembly, 5 ORFs are missing and 4 ORFs are aligned with low-score, despite the better R50 value of HHHH than MSSH. The failure is caused by the inclusion of the SOLiD data at the unitig stage, where 7 ORFs are missing in the Hxxx unitigs, even though the percentage of the identically aligned ORFs are increased to 97.8% from Mxxx. We found that these missing ORFs, as well as the low-score ORFs in HHHH, are located on the mitochondrial chromosome. The mitochondrial chromosome is known to have high AT content (74% in *A. oryzae*), and the SOLiD short reads become erroneous due to the AT bias in the SOLiD system [30]. Indeed, 7 missing ORFs in the denovo2 assembly are of mitochondria, too.

Although the SOLiD data are problematic in the assemblies of AT-rich regions at the unitig stage, these data are useful in the remainder of the assembly stages. While the mitochondrial ORFs are distributed over 4 strands in the MMMM assembly, all of the 16 ORFs are united on a single strand in the MSSH assembly. Note, however, that on the single strand, two genes (AO090002000060 and AO090002000070) are found to be swapped. In the Mxxx unitigs, these two genes form a single unitig, which seems to be erroneously scaffolded to construct a single strand.

**Reproducibility of SMB gene clusters** The number of SMB gene clusters found in the assembled strands is summarized in Table 6. Among 75 SMB clusters, up to 70 clusters can be located on respective single MSSH strands, with 58 clusters identically aligned. The average length of the SMB gene clusters is 24 kbp, with the maximum length of 56 kbp. Even though the MSSH R50 value of 210 kbp is well beyond the SMB sizes, 5 clusters are left unaligned on a single strand. This indicates the difficulties to reproduce whole SMB clusters from short read data.

**Table 6:** Number of SMB gene clusters reproduced in the assemblies.

	nucleotide sequence				ORF sequence		
mode	identical	single^a^	double^a^	missing	single^b^	double^b^	missing
*A. oryzae*	(75)^c^						
HHHH	60	9	0	6	73	2	0
MSSH	58	12	0	5	73	2	0
MMMM	45	5	4	21	53	21	1
SSSS	6	24	3	42	61	10	4
denovo2	16	35	0	24	73	2	0
*S. avermitilis*	(37)^c^						
HHHH	25	7	0	5	34	1	2
MSSH	25	7	0	5	33	2	2
MMMM	25	6	0	6	33	1	3
SSSS	0	0	0	37	6	5	26
denovo2	0	1	0	36	19	4	14

^a^ More than 99.9% of the nucleotide sequence are reproduced on single/double strands.

^b^ All ORFs are aligned orderly on single/double strands.

^c^ Total number of SMB gene clusters.

If we can ignore inter-gene regions and focus on the relative arrangements of the ORFs, 73 out of 75 SMB gene clusters are located on the respective MSSH strands. Still, two clusters, denoted as AO090001000293 and AO090020000527 in [28], are not located on a single strand, but are split on two strands. These clusters have a region few kbp in length with high AT content, which divides the ORFs into two groups. We suspect that the SOLiD mate-paired reads are missing in the AT rich region, leaving the two strands unconnected. Looking only at the ORFs, the denovo2 assembly reproduces the same 73 SMB gene clusters as MSSH, supporting the findings in our previous report [7], whereas the MMMM assembly only reproduces 53 clusters. With respect to the nucleotide sequence, however, only 16 out of 73 clusters were identically aligned in the denovo2 assembly, while 45 out of 53 clusters were identically aligned in the MMMM assembly. These observations indicate that the SOLiD mate-paired reads with long insertion length are indispensable when stitching ORFs together to form a SMB gene cluster, while the MiSeq data with long read length are essential to reproduce nucleotide sequences accurately.

### *Streptomyces avermitilis* MA-4680

In the previous section, the performance of the de novo assemblies from short read data was shown to be improved by using SOLiD mate-paired data in conjunction with MiSeq data. It was also anticipated that the hybrid scheme may not work well for genomes with biased nucleotide composition, because SOLiD data are unreliable when generated from such genomes [30]. To investigate this possibility, the hybrid scheme is applied on *S. avermitilis*, whose genome is known to have high GC content (71%).

The base information of the input short read data for *S. avermitilis* assemblies is summarized in Table 1. The SOLiD data were down-sampled at *N*_lowQ_ = 6, statistics of which is also listed in Table 1. Because of the low quality of the SOLiD data, only 12% of the mate-pairs remained after the down-sample, even though the Exact Call Chemistry (ECC) module [25] was employed. This is in contrast to the *A. oryzae* case, where 78% remained even at *N*_lowQ_ = 0. The reference sequences [29] and a set of 7683 ORFs are obtained from [31]. The location of the SMB gene clusters were taken from [32], where 37 candidates are listed.

**Fig 3:**
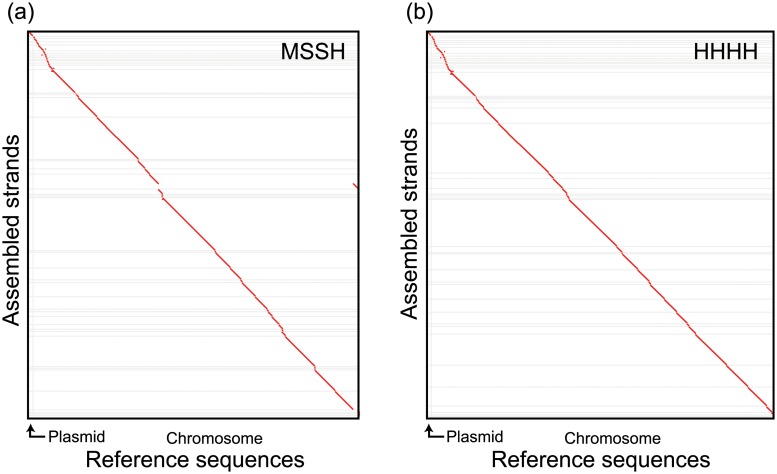
Dotplot alignments of assembled strands against the reference genome sequence of *S. avermitilis*. Alignments shorter than 4000 bp were omitted from the plots. Forward and reverse alignments are plotted in red and blue colors, respectively. (a) The MSSH assembly, (b) the HHHH assembly.

Characteristic indices of the assemblies are summarized in Table 2. Apparently, the SOLiD only assemblies (SSSS and denovo2) are not successful. Even though the coverage of the SOLiD data is about 100 and is very similar to the *A. oryzae* case, the N50 and R50 values are reduced by a factor of 30. On the contrary, these indices for the MMMM assembly improved by a factor of 1.5, probably due to the quadrupled coverage of the MiSeq data. Therefore, it was unexpected to observe the improved performance of the hybrid assemblies, where the incorporation of the SOLiD data enhances the R50 value by a factor of 1.5 ∼ 2 from MMMM. It was also noted that the performance of the HHHH assembly is much improved from MSSH. Detailed analysis showed that the hybrid data should be applied both at the unitig and the contig/scaffolding stages to increase the R50 value by 50 kbp. The superiority of HHHH over MSSH was also confirmed by the dotplots shown in Fig 3, where a major misjoin in MSSH disappears in the HHHH assembly. Note that the *S. avermitilis* genome comprises of a single linear chromosome and a plasmid.

The number of ORFs reproduced in the assembled strands is summarized in Table 5. As in the *A. oryzae* case, the best reproducibility of the gene-coding regions was obtained using the MiSeq data: 93.2% of the ORFs are already revealed identically in the Mxxx unitigs, which yields high reproducibility of 95.6% in the hybrid assemblies. Meanwhile, only 21% of the ORFs can be aligned identically and more than 100 ORFs are missing in the denovo2 assembly, reflecting the low quality of the SOLiD short reads. Combining the SOLiD data with the MiSeq data, however, does not induce misassemblings of the ORFs in the *S. avermitilis* case. We suspect that the coverage of the SOLiD data is uneven over the genome, and the read parts are fairly accurate due to the ECC module.

The reproducibility of the SMB gene clusters is almost parallel to the ORF reproducibility. The number of SMB gene clusters aligned on the assembled strands is shown in Table 6. In the hybrid assemblies, 32 clusters out of 37 are aligned on respective single strands, with 25 clusters identically aligned. These 25 clusters are, however, already found in the Mxxx unitigs, indicating that the long insertion length of the SOLiD data is not exploited for stitching ORFs together. If we focus only on the relative arrangement of ORFs ignoring inter-gene sequences, three clusters are still not aligned on a single strand in the HHHH assembly: two clusters (No. 7 and 20 in [32]) are scattered over 5 strands, and one cluster (No. 4) are split into 2 strands. While the average length of the SMB clusters are 16 kbp, these 3 clusters are longer than 80 kbp and the three largest ones in *S. avermitilis*. This also suggests that a reduced amount of long-range bridging information from the SOLiD data is available, at least for the SMB regions.

## Conclusion

A hybrid assembly, where MiSeq and SOLiD short read data are used in combination, was shown to be effective for fungal genome assemblies. These two types of data work complementarily to improve the overall genome analysis. The accuracy of the nucleotide sequences is achieved by the long read length of MiSeq data, while the long-range arrangement of sequences is supported by the long insertion length of the SOLiD mate-paired reads. Although emerging NGS platforms like MiSeq are cost-effective in gene sequencing, assemblies based solely on them are less reliable for practical genome analysis such as the identification of SMB gene clusters. By coupling NGS outputs of different kinds, sufficiently long nucleotide sequences can be reproduced for practical analysis, even if only short read data are available.

Because the AT bias of the SOLiD short reads are erroneous at regions where the AT/GC balance are highly biased. Inclusion of such erroneous data were found to skew the de Bruijn assembly, so that the MiSeq only assembly is recommended at the unitig stage, if the SOLiD data are taken without the Exact Call Chemistry module. At present, the SOLiD data are converted to the base space before being used in the assembly, so that a single read error on a short read makes the subsequent part of the read unusable, limiting the availability of the SOLiD data. For future work, the performance of the pipeline will be improved possibly by mapping SOLiD color space reads directly onto the base-space unitigs/contigs at the contig/scaffold stages. The use of the MiSeq mate-pair kit (which was not available at the time of the experiment) will be helpful as well, because the MiSeq data are free from the AT bias and the conversion errors. The performance of the present pipeline can also be assessed by comparing with other assemblers like Allpaths-LG [33, 34] based on the same MiSeq data set.
